# Comparative analysis of the analgesic effects of intercostal nerve block, ultrasound-guided paravertebral nerve block, and epidural block following single-port thoracoscopic lung surgery

**DOI:** 10.1186/s13019-024-02877-7

**Published:** 2024-07-01

**Authors:** Liang Shen, Zi Ye, Fei Wang, Gao-Feng Sun, Cheng Ji

**Affiliations:** 1 Department of Thoracic Surgery, The Second Affiliated Hospital of Nantong University, Nantong First People’s Hospital, No. 666 of Shengli Road, Chongchuan District, Nantong, 226000 China; 2 Department of Neurosurgery, Nantong First People’s Hospital, The Second Affiliated Hospital of Nantong University, Nantong, 226000 People’s Republic of China

**Keywords:** Analgesia, Epidural block, Intercostal nerve block, Paravertebral nerve block, Thoracoscopy

## Abstract

**Objective:**

In this study, we compared the analgesic effects of intercostal nerve block (ICNB), ultrasound-guided paravertebral nerve block (PVB), and epidural block (EB) following single-port thoracoscopic lung surgery.

**Method:**

A total of 120 patients who underwent single-hole thoracoscopic lung surgery were randomly and equally divided into three groups: ICNB group, the PVB group, and the EB group. ICNB was performed under direct thoracoscopic visualization before the conclusion of the surgery in the ICNB group, while PVB and EB were performed after general anesthesia in the PVB and EB groups, respectively. Patient-controlled intravenous analgesia (PCIA) was used following the surgery in all the groups. The following indicators were recorded: Intraoperative sufentanil dosage, anesthesia awakening time, postoperative intubation time, nerve block operation time, postoperative visual analog scale (VAS) pain scores during resting and coughing at regular intervals of 0, 2, 4, 8, 24, and 48 h, the time until first PCIA, number of effective compressions within 24 h postoperatively, number of rescue analgesia interventions, and the side effects.

**Results:**

In comparison to the ICNB group, the PVB and EB groups had a lower intraoperative sufentanil dosage, significantly shorter anesthesia awakening time, and postoperative intubation time, but longer nerve block operation time, lower VAS scores when resting and coughing within 24 h postoperatively (all p-values less than 0.05). Conversely, there were no statistically significant differences in VAS scores during resting and coughing after 24 h (all p-values greater than 0.05). Time to first PCIA, number of effective compressions and number of rescue analgesia at the 24-hour mark postoperatively were significantly better in the PVB and EB groups than that in the ICNB group (*P* < 0.05). However, there was a higher incidence of side effects observed in the EB group (*P* < 0.05).

**Conclusion:**

The analgesic effect of PVB and EB following single-port thoracoscopic lung surgery is better than that of ICNB. PVB causes fewer side effects and complications and is safer and more effective.

## Introduction

Lung cancer is currently one of the most common malignant tumors worldwide [1], and surgical resection is the most effective form of treatment for lung cancer in the early stages [2]. The widespread use of televised thoracoscopic techniques, especially those involving uniportal video-assisted thoracoscopic surgery (UVATS) techniques, has the potential to mitigate the extent of injury inflicted upon the chest wall muscles and nerves, hence minimizing the surgical stress experienced by patients. However, patients continue to experience moderate to severe pain following surgery due to irritation from chest tubes, particularly within the initial 24-hour postoperative timeframe [3]. Inadequate postoperative analgesia can prevent patients from expectorating sputum effectively, thus increasing the risk of respiratory complications and affecting postoperative recovery. Currently, there is a lack of consensus on the optimal technique for analgesia following thoracoscopic surgery. A variety of analgesic strategies and techniques have become the focus of growing research efforts [4]. Epidural anesthesia, initially advocated for effective pain management following thoracic surgery, was associated with a notable incidence of side effects and potential complications. In recent years, regional anesthesia techniques have gained widespread application in clinical practice due to their capacity to diminish postoperative pain and reduce opioid usage. Consequently, the pursuit of the most optimal regional anesthesia technique is anticipated to yield the most effective, safe, and straightforward form of post-thoracoscopic analgesia.

Hence, the present study was devised with the aim of conducting a comparative analysis on the analgesic effects of intercostal nerve block (ICNB), ultrasound-guided paravertebral nerve block (PVB), and epidural block (EB) following single-port thoracoscopic lung surgery (hereafter referred to as ICNB, PVB, and EB), and to evaluate the effectiveness of these three techniques.

## Study data and method

### General information

This study was a prospective, randomized controlled study, and was approved by the Medical Ethics Committee of the First People’s Hospital of Nantong City. The study sample consisted of 120 participants who underwent single-port thoracoscopic lung surgery in the thoracic surgery department of the hospital between October 2020 and January 2022. Among these participants, 90 cases were of pulmonary lobectomy, 9 cases of segmentectomy, and 21 cases of pulmonary wedge resection. All participants signed a written informed consent and were randomly grouped. The inclusion criteria for this study are as follows: (1) Age between 20 and 75 years. (2) Preoperative ASA classification class I-II. (3) BMI less than 30 kg/m^2^. (4) Patients gave informed consent and were willing to participate in this study. The exclusion criteria for this study are as follows: (1) Individuals allergic to anesthetics. (2) History of coagulation disorders and severe infections. (3) Individuals with severe cardiac disease, hepatic and renal insufficiency. (4) Individuals with chronic pain, psychiatric disorders, and long-term use of opioids. (5) Individuals who require conversion to open surgery during the procedure. (6) Individuals with a preoperative ASA classification of III or higher. During the preoperative visit, patients were instructed on how to use a patient-controlled intravenous analgesia (PCIA) device for pain management and how to use a visual analog scale (VAS) to assess pain levels during periods of resting and coughing.

### Anesthesia and surgery

The patients underwent continuous surveillance for electrocardiographic activity, oxygen saturation levels, and respiratory rate. Additionally, the radial artery was catheterized to facilitate invasive monitoring of arterial pressure. General anesthesia was induced with 0.5 µg/kg sufentanil, 1.5–2.0 mg/kg propofol, and 0.2 mg/kg atracurium. Fiber optic bronchoscope-guided double-lumen tracheal intubation was performed. Intraoperatively, the patients were administered 1.5% sevoflurane through inhalation. Additionally, intravenous infusions of dexmedetomidine and sufentanil were administered at the rate of 0.5 µg/kg/hour. The dosages of these drugs were adjusted according to the level of anesthesia and blood pressure, which was monitored using the Bispecteral Index (BIS). Tidal volume was adjusted to 6 ml/kg, and respiratory rate at 12 to 15 breaths per minute. Unilateral lung ventilation was performed while preparing for the surgery. The ventilation frequency and tidal volume were adjusted to maintain end-tidal carbon dioxide pressure (PetCO_2_) at 35–45 mmHg. All surgical procedures were performed with a 3–4 cm incision at the fifth intercostal space between the anterior and midaxillary lines.

Regional nerve block: Ultrasound-guided paravertebral nerve block: A high-frequency ultrasound probe was used to scan about 2–2.5 cm from the T4-5 spinous process, clearly displaying the transverse process, pleura, and thoracic paraspinal space. After the puncture needle was inserted vertically into the skin from the outer side of the probe, the direction of insertion was adjusted. The needle penetrated the superior costotransverse ligament, successfully accessing the paravertebral space. Following verification of the absence of blood or cerebrospinal fluid reflux, an injection comprising 20 ml of 0.375% ropivacaine hydrochloride was administered into the paravertebral space at the T4-5 level.

Epidural block: Epidural block was performed in the lateral position before induction of anaesthesia. Epidural block was performed on the median T5 supraspinal puncture point as the anatomic marker. A solution containing 0.5% lidocaine in a 3 ml volume was injected to induce anesthetize intradermally, subcutaneously, and deeply, extending up to the supraspinous and interspinous ligaments. The direction for needle insertion in preparation for epidural puncture was subsequently determined. The skin and ligaments were punctured with a 15G sharp needle. The epidural puncture needle was introduced into the skin with careful alignment, traversing through the supraspinous ligament, interspinous ligament, and ligamentum flavum until a distinct sensation of descent or a positive outcome in the negative pressure test was discerned. These indicators signify the successful advancement of the needle tip into the epidural space. A catheter for epidural anesthesia was inserted at a depth of 3–5 cm and securely positioned. A test volume of 3 ml of ropivacaine hydrochloride with a concentration of 0.375% was administered through a catheter. During the surgical procedure, ropivacaine was administered through continuous infusion at a rate of 4 to 5 ml/hour. A loading dose of 2 mg of morphine was administered prior to the conclusion of the surgery. The epidural catheter was removed at the end of the surgical procedure.

Intercostal nerve block: Prior to concluding the surgical procedure, the intercostal nerve was blocked by injecting 4 ml of ropivacaine hydrochloride at a concentration of 0.375%. The drug was injected into each intercostal space at the 3rd to 7th intercostal space on the surgical side at a distance of 5–8 cm from the outer side of the costovertebral joint.

Postoperatively, the administration of patient-controlled intravenous analgesia (PCIA) was prescribed. The analgesic pump was configured as follows: A solution was prepared by diluting 100 µg sufentanil and 10 mg of azasetron in saline to a final volume of 100 ml. The analgesic pump infusion rate was set to a background dose of 1 mg/hour, a single dose of 2 ml, and a lockout time of 15 min. Patients were instructed to assess the pain degree using the VAS, in which 0 means no pain, 1–3 indicates mild pain that does not affect work and daily activities, 4–7 indicates moderate pain that affects work but not daily activities, and 7–10 indicates severe pain that significantly affects both work and daily activities. If the VAS scores were greater than or equal to 4 at rest or when coughing, the patient can activate the analgesic pump by pressing the designated button. The analgesic effect was considered to be poor if the VAS score was still greater than 4 after 2 consecutive presses. In such cases, a dose of 30 mg of Ketorolac tramine was administered intramuscularly as a means of providing rescue analgesia.

### Primary observational indicators

The following indicators were documented in the study: Intraoperative sufentanil dosage, anesthesia awakening time, postoperative intubation time, nerve block operation time, postoperative VAS scores when resting and coughing at various time intervals (0, 2, 4, 8, 24, and 48 h), time until first PCIA was administered, number of effective compressions within 24 h postoperatively, number of rescue analgesia interventions, and side effects. Anesthesia awakening time refers to the time from withdrawal of sedation and analgesic drugs to full consciousness after surgery. Postoperative intubation time is from the end of the surgery to the removal of the tracheal tube. Nerve block time refers to the time from the beginning to the end of the nerve block operation.

### Statistical analysis

SPSS 19.0 statistical software was used to analyze the data. The Kolmogorov-Smirnov test was used to determine whether the data satisfied a normal distribution. The measured data was expressed as mean ± standard deviation ($\stackrel{-}{x}\pm s$), and the *t*-test was used for the comparison between groups. Rank sum test was used for the comparison between groups of non-normally distributed data. The difference was considered statistically significant if *P* < 0.05.

## Results

### Comparison of general data

**Fig. 1:**
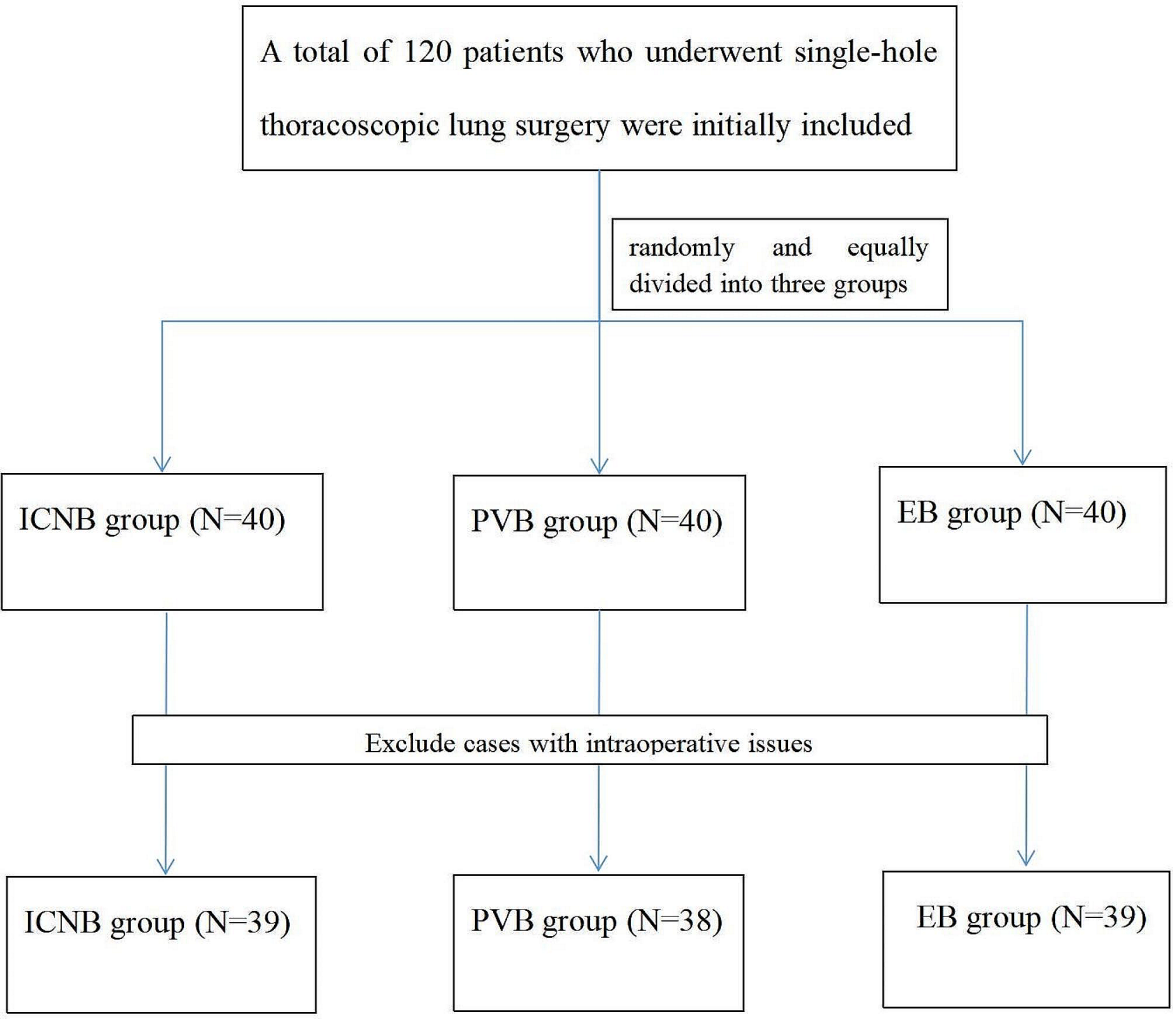
The consort flow chart of the study. Note: intercostal nerve block (ICNB), ultrasound-guided paravertebral nerve block (PVB), epidural block (EB)

A total of 120 eligible patients were initially included in the study, with 40 patients in each group. Among them, there was one case of intra-operative conversion to open surgery in the ICNB group, two cases of midway withdrawal in the PVB group, and one case of midway withdrawal in the EB group. Finally, 116 patients were included in the study, among which 39 patients were in the ICNB group, 38 patients were in the PVB group, and 39 patients were in the EB group. There was no statistically significant difference in the general information of the patients (*P* > 0.05). See Table 1; Fig. 1 for details.

**Table 1:** Comparison of general information between the three groups

Indicators	ICNB group (*n* = 39)	TPVB group (*n* = 38)	EB group (*n* = 39)	*P*-value
Gender (n, %)				
Male	21(53.8%)	25(65.8%)	20(51.3%)	0.602
Female	18(46.2%)	13(34,2%)	19(48.7%)	
Age (year)	61.6 ± 10.4	64.1 ± 7.0	63.3 ± 11.6	0.630
BMI (Kg/m^2^)	22.9 ± 2.6	23.5 ± 2.4	22.9 ± 3.1	0.532
ASA classification				
Class I	16(41.0%)	18(47.4%)	17(43.6%)	0.488
Class II	23(59.0%)	20(52.6%)	22(56.4%)	
Diabetes	3(7.7%)	6(15.8%)	4(10.3%)	0.724
Hypertension	4(10.3%)	7(18.4%)	5(12.8%)	0.671

### Comparison of perioperative related indicators

There was no significant difference between the three groups of patients in terms of operating time, surgical procedure, intraoperative bleeding, and postoperative chest tube intubation duration (*P* > 0.05). The intraoperative sufentanil dosages of the PVB and EB groups were less than that of the ICNB group (*P* < 0.05), and anesthesia awakening time and postoperative intubation time were also significantly shorter than that of the ICNB group (*P* < 0.05). The operating time of ultrasound-guided paravertebral nerve block and epidural block was significantly longer than that of the intercostal nerve block (*P* < 0.05), see Table 2.

**Table 2:** Comparison of perioperative related indicators between the three groups

Indicators	ICNB group (*n* = 39)	TPVB group (*n* = 38)	EB group (*n* = 39)	*P*-value
Operation time (min)	118.4 ± 38.2	124.8 ± 46.0	124.5 ± 43.1	0.522
Surgical procedure				
Pulmonary lobectomy	28(71.8%)	31(81.6%)	29(74.4%)	
Segmentectomy	3(7.7%)	2(5.3%)	4(10.3%)	0.524
Pulmonary wedge resection	8(20.5%)	5(13.1%)	6(15.3%)	
Intraoperative dosage of sufentanil (µg)	41.9 ± 3.6	32.7 ± 5.7	33.86 ± 2.8	0.016
Nerve block time (min)	3.6 ± 0.6	12.1 ± 0.5	15.3 ± 0.3	0.024
Bleeding volume (ml)	121.5 ± 82.6	133.9 ± 94.2	139.3 ± 91.2	0.856
Anesthesia awaking time (min)	25.8 ± 4.1	19.0 ± 3.4	19.5 ± 4.3	0.002
Postoperative intubation time (min)	31.2 ± 4.9	23.6 ± 4.2	24.6 ± 4.2	0.034
Chest tube intubation time (d)	3.6 ± 0.4	3.3 ± 0.3	3.5 ± 0.3	0.329

### Comparison of postoperative VAS scores and analgesic indicators

**Fig. 2:**
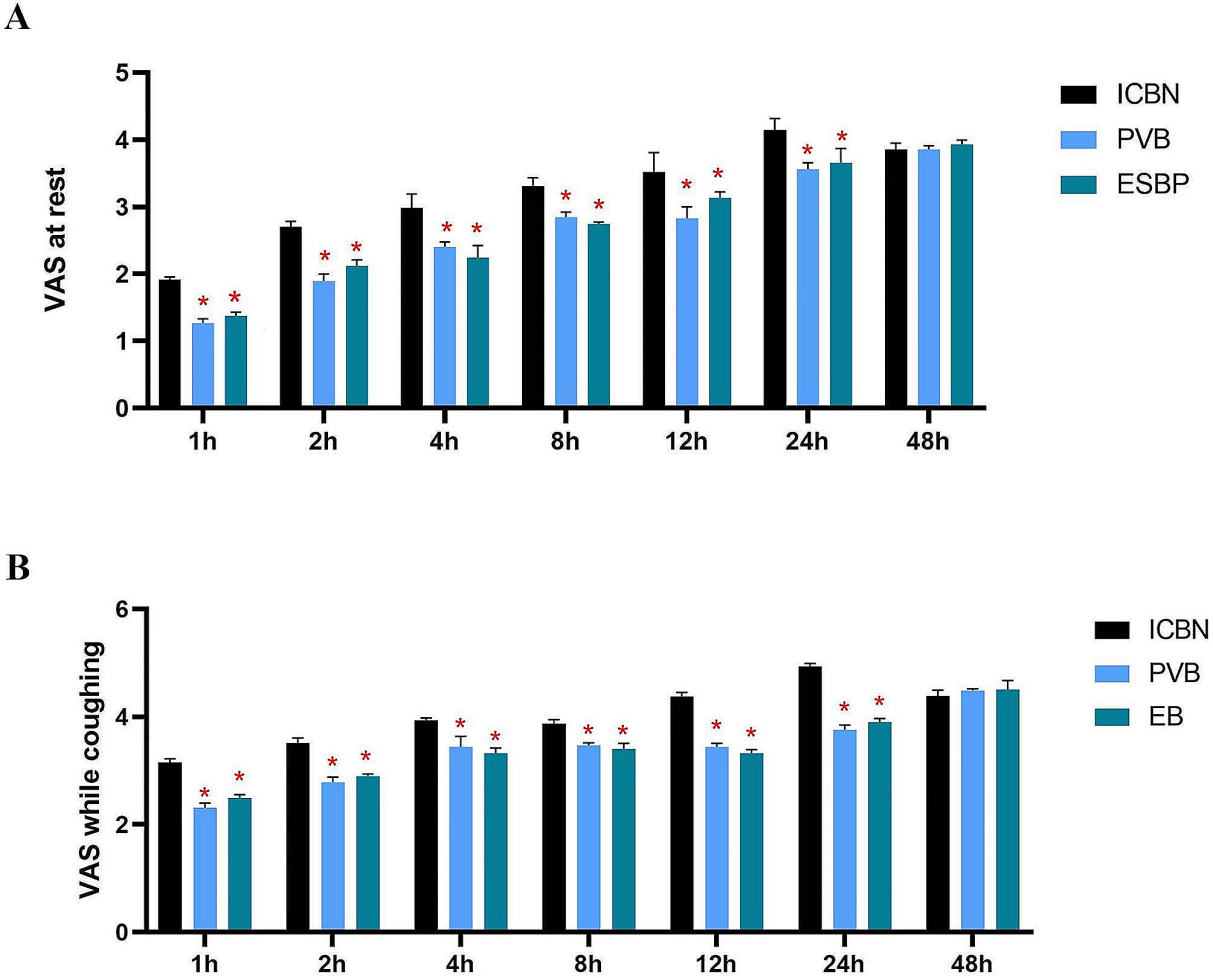
Postoperative VAS scores when resting (A) and coughing (B) were lower in the PVB and EB groups compared to the ICNB group at all time points during the 24-hour period (*P* < 0.05), however, there was no significant difference after 24 h (*P* > 0.05). Note: intercostal nerve block (ICNB), ultrasound-guided paravertebral nerve block (PVB), epidural block (EB), * *P* < 0.05

Postoperative VAS scores when resting and coughing were lower in the PVB and EB groups than those in the ICNB group at all time points during the 24-hour period (*P* < 0.05), but there was no significant difference after 24 h (*P* > 0.05), see Fig. 2. The time to first use of PCIA was shorter in the PVB and EB groups than that in the ICNB group (*P* < 0.05). The number of effective PCIA attempts and rescue analgesia within the 24 h postoperatively were lower in the PVB and EB groups than those in the ICNB group (*P* < 0.05), as shown in Table 3.

**Table 3:** Comparison of postoperative analgesic indicators between the three groups

Indicators	ICNB group	TPVB group	EB group	*P*-value
Time to first PCIA (h)	9.8 ± 1.9	12.5 ± 2.1	13.4 ± 2.3	0.021
Numbers of effective compressions within 24 h after surgery	4.0 ± 1.2	2.0 ± 1.0	2.0 ± 1.5	0.018
Numbers of postoperative rescue analgesia interventions	2.0 ± 0.6	1.0 ± 0.4	1.0 ± 0.3	0.042

### Comparison of postoperative side effects and complications

In the three groups, the incidences of postoperative complications such as dizziness, headache, nausea, vomiting, urinary retention, and hypoxemia were significantly higher in the EB group than those in the ICNB group and the PVB group (*P* < 0.05). However, there was no significant difference between the three groups in terms of postoperative pulmonary infections and duration of hospitalization (*P* > 0.05), as shown in Table 4.

**Table 4:** Comparison of postoperative side effects and complications between the three groups

Indicators	ICNB group (*n* = 40)	TPVB group (*n* = 40)	EB group (*n* = 40)	*P*-value
Dizziness, headache	3	2	6	0.041
Nausea and vomiting	2	2	5	0.006
Urinary retention	1	2	4	0.028
Hypoxemia	1	1	5	0.015
Lung infection	1	0	1	0.825
Postoperative Hospitalization length (d)	4.6 ± 0.5	4.3 ± 0.8	5.1 ± 0.4	0.272

## Discussion

This study evaluated the analgesic effects of ICNB, PVB, and EB in single-port thoracoscopic lung surgery. The results showed that the analgesic effect of PVB and EB was better than that of ICNB. In addition, PVB was related to fewer side effects and complications than the other two methods. These findings indicated that PVB might be the most suitable for pain control after single-port thoracoscopic lung surgery.

Pain following thoracic surgery is strongly associated with pulmonary complications such as lung infections and respiratory failure. This association is especially pronounced in the elderly, smokers, and those with moderate to severe chronic obstructive pulmonary disease [5]. The presence of wound pain may result in difficulty in coughing up sputum, shallow breathing, atelectasis, and reduced lung capacity. These complications can subsequently lead to imbalances in ventilation-blood flow ratio, hypoxemia, and possibly even right heart failure. These complications can prolong the duration of intensive care and hospitalization, and as well as increase the risk of mortality during the perioperative period [6–8]. Although systemic opioids have been the mainstay of treatment for thoracic postoperative pain, they are associated with side effects such as nausea, vomiting, respiratory depression, and urinary retention. The alteration of the administration route of opioids, both independently or in combination with local anesthetic drugs can improve analgesic effects and reduce side effects [9–11]. The selection of regional anesthetic techniques such as erector spinae plane block, intercostal nerve block, paravertebral nerve block, and epidural block, serves not only to proficiently alleviate post-thoracoscopic pain but also contributes to a reduction in opioid dosage, consequently mitigating associated side effects.

Epidural anesthesia was first recommended for management of perioperative analgesia in thoracic surgery [12, 13], and is the preferred technique for postoperative analgesia in thoracic surgery. Epidural anesthesia is usually administered as a combination of opioids and local anesthetics. Epidural opioids and local anesthetics provide superior analgesia and reduce respiratory complications such as pulmonary atelectasis and lung infections in comparison to systemic opioids [14]. The administration of local anesthetics as a standalone method for pain control often requires higher concentrations and dosages with potentially more side effects [15]. Typically, local anesthetics exhibit a sequential blockade of nerves, affecting autonomic nerves initially, followed by sensory nerves, and ultimately motor fibers. Ideally, the administration of local anesthetics is tailored to achieve a precise concentration for selective sensory block while minimizing impact on motor fibers. However, as the concentration of local anesthetics increases in clinical practice, so does the risk of impairment of motor fibers. The combined usage of opioids and local anesthetics, although having a better nerve blocking effect, causes a significantly higher incidence of gastrointestinal reactions such as nausea and vomiting. In this study, despite the administration of a higher dosage of opioids in the ICNB group for postoperative intravenous analgesia, the incidences of side effects associated with postoperative headache and dizziness, nausea, vomiting and postoperative urinary retention were still significantly higher in the EB group compared to both the ICNB and PVB groups. Epidural anesthesia techniques may present heightened challenges or, in severe instances, prove unsuccessful in scenarios characterized by pronounced spinal deformities, neurological disorders, or variations in thoracic vertebral anatomy. Additionally, it is contraindicated in cases of sepsis and coagulation disorders. Epidural puncture can also result in complications like cerebrospinal fluid leakage, epidural hematoma, epidural infection, and spinal cord injuries [16]. 

Ultrasound-guided paravertebral nerve block entails the administration of local anesthetic into the thoracic paravertebral space with the aid of ultrasound imaging. This allows for the precise placement of the injection, enabling the diffusion of the local anesthetic into the surrounding paravertebral space, intercostal space, and epidural space. Consequently, this technique effectively blocks the branches of the thoracic spinal nerves sympathetic nerves [17, 18], and can provide analgesia comparable to thoracic segmental epidural block, but with fewer side effects. Complications such as postoperative urinary retention, nausea, vomiting, hypotension, and pulmonary infection are less likely to occur with ultrasound-guided paravertebral nerve block [19]. The findings of this study indicate that the utilization of preoperative ultrasound-guided paravertebral nerve block reduced the consumption of intraoperative sufentanil and shortened the time of anesthetic awakening and intubation. These results suggest that the combination of general anesthesia and ultrasound-guided paravertebral nerve block can provide effective intraoperative analgesia and expedite the recovery from an anesthetic state for thoracoscopic lung surgery. In addition, Chen et al., discovered that ultrasound-guided paravertebral nerve block provided superior analgesia following thoracoscopic surgery in comparison with ultrasound-guided erector spinae plane block [20]. Presently, the application of ultrasound-guided paravertebral nerve block is predominantly constrained due to its reliance on ultrasound-guided puncture, a procedure characterized by complexity and associated risks, including the potential for local hematoma, severe hypotension, and pneumothorax [19]. 

Intercostal nerve block has also been used effectively for postoperative pain management in thoracic surgery. Additionally, a percutaneous preoperative intercostal nerve block can be administered with the assistance of ultrasound guidance to successfully block the intercostal nerve in proximity to the surgical incision site [21]. Toledo-Pereyra et al., conducted a prospective randomized controlled trial comparing intercostal nerve block in the fifth intercostal space with no treatment, and discovered significantly better postoperative expiratory volume in one second (FEV1), forced vital capacity (FVC), and pain management in the intercostal nerve block group [22]. The most common complications associated with intercostal nerve block include general spinal anesthesia. Furthermore, there have been documented instances of hypotension, as well as cardiac and central nervous system complications arising from the systemic absorption of local anesthetics [23]. In addition, the duration of analgesic effect provided by intercostal nerve block is limited to a period of 6 to 8 h. Consequently, it requires repeated administrations or the use of systemic opioids to sustain postoperative analgesia. It should be noted that relying on intercostal nerve block is insufficient for adequate management of postoperative pain. In this study, the administration of nerve blocks targeting the incision site, as well as the upper and lower intercostal areas, was carried out with direct visualization toward the culmination of the thoracoscopy procedure. This approach was found to be simple to execute. The operating time of the nerve block was significantly shorter than that of epidural anesthesia and ultrasound-guided paravertebral block. The administration of intercostal nerve block at the culmination of the thoracoscopy procedure did not result in a reduction in intraopioid dosages. Additionally, the duration of awakening and intubation time at the end of the procedure were longer when compared to the groups that received paravertebral and epidural blocks. The intercostal nerve block had a good analgesic effect for a short duration following surgery. While the analgesic efficacy of the intercostal nerve block was comparatively less potent than that of ultrasound-guided paravertebral nerve block and epidural anesthesia, its administration proved to be straightforward under direct vision. The incidence of complications was lower. Notably, this technique demonstrated effectiveness in mitigating postoperative pain on the first day. Consequently, the intercostal nerve block holds promise for favorable clinical application.

Based on the results of this study, the pain scores in the group receiving intercostal nerve block were significantly higher compared to those in the groups receiving ultrasound-guided paravertebral nerve block and epidural block within the first 24 h following surgery. Furthermore, ultrasound-guided paravertebral nerve block and epidural anesthesia provided superior analgesic effect compared to intercostal nerve block for thoracoscopic surgery. However, the discernible difference in analgesic efficacy was not notably significant, and this observation may be attributed to the following reasons. In the context of thoracoscopic surgery, especially when single-port thoracoscopic surgery is performed, the surgical incision is typically limited to a length of 3 to 4 cm. This restricted incision primarily impacts the nerves and muscles within a single intercostal space, thereby reducing the intensity of postoperative pain resulting from the incision. Another significant factor contributing to post-surgical incision is the irritation caused by the presence of a chest tube. Advancements in surgical procedures have led to a reduction in the duration of post-surgical air leakage, thereby enabling the early removal of chest tubes. This intervention has been found to alleviate postsurgical pain. In the context of highly invasive procedures like open chest surgery, wherein the intensity of postoperative pain is more severe, the varying efficacy of different regional nerve blocks is more pronounced. In addition, patients in China do not realize the importance of effective pain management and perceive that postoperative pain is an inevitable consequence of surgery. As a result, patients rarely engage the PCIA button despite experiencing severe pain. This behavior serves to diminish the differences between different regional block modalities [24, 25]. 

There are several limitations in the present study. This study constitutes a single-center, small sample, exploratory clinical trial, and further multicenter, large-sample clinical trials are required to further confirm this conclusion. Moreover, the surgical procedure used in this study was a single-port thoracoscopic surgery. It is important to acknowledge that other conclusions may be drawn if the surgical approach were to be modified to either three-port thoracoscopic surgery or open thoracic surgery. Therefore, it is imperative to conduct a comparative analysis of the analgesic efficacy exhibited by various analgesic modalities across other surgical interventions.

## Conclusion

In summary, the intercostal nerve block procedure, although simple and safe, has poor analgesic effect. Conversely, the epidural block technique results in good analgesic effect, but its operational process is complicated with many complications and side effects. Ultrasound-guided thoracic paravertebral block not only yields efficient pain management, but also results in less administration of analgesic drugs throughout the preoperative period. This approach has the potential to be utilized for the purpose of pain control subsequent to the thoracic surgical procedures.

## Data Availability

No datasets were generated or analysed during the current study.
